# The Cyanobacteria Derived Toxin Beta-N-Methylamino-L-Alanine and Amyotrophic Lateral Sclerosis

**DOI:** 10.3390/toxins2122837

**Published:** 2010-12-20

**Authors:** Sandra Anne Banack, Tracie A. Caller, Elijah W. Stommel

**Affiliations:** 1 The Institute for Ethnomedicine/PO Box 3464, Jackson, WY 83001, USA; 2 Department of Neurology, Dartmouth Hitchcock Medical Center/Lebanon, NH 03756, USA; Email: Tracie.A.Caller@Hitchcock.org (T.A.C.); Elijah.W.Stommel@Hitchcock.org (E.W.S.)

**Keywords:** Amyotrophic lateral sclerosis, Alzheimer’s disease, cyanobacteria, Beta-N-methylamino-L-alanine, BMAA, epidemiology

## Abstract

There is mounting evidence to suggest that environmental factors play a major role in the development of neurodegenerative diseases like ALS (Amyotrophic Lateral Sclerosis). The non-protein amino acid beta-N-methylamino-L-alanine (BMAA) was first associated with the high incidence of Amyotrophic Lateral Sclerosis/Parkinsonism Dementia Complex (ALS/PDC) in Guam, and has been implicated as a potential environmental factor in ALS, Alzheimer’s disease, and other neurodegenerative diseases. BMAA has a number of toxic effects on motor neurons including direct agonist action on NMDA and AMPA receptors, induction of oxidative stress, and depletion of glutathione. As a non-protein amino acid, there is also the strong possibility that BMAA could cause intraneuronal protein misfolding, the hallmark of neurodegeneration. While an animal model for BMAA-induced ALS is lacking, there is substantial evidence to support a link between this toxin and ALS. The ramifications of discovering an environmental trigger for ALS are enormous. In this article, we discuss the history, ecology, pharmacology and clinical ramifications of this ubiquitous, cyanobacteria-derived toxin.

## 1. Introduction

Amyotrophic Lateral Sclerosis (ALS) is a debilitating and fatal neuromuscular disease with an average annual incidence worldwide of 2 per 100,000. Approximately 8–10% of all cases are familial, about half of which are characterized by superoxide dismutase mutation (SOD-1). The other 90–92% of ALS cases occur sporadically, with no known familial history. At present there is no known cause for sporadic ALS, though a number of environmental compounds have been implicated as potential etiological agents. The most documented and relatively well established toxicological risk factor for ALS is smoking [1,2,3,4,5,6,7]. Other associations that have been proposed are occupational exposure to electromagnetic radiation [8,9], contact with pesticides and heavy metals such as lead and mercury [10,11,12,13,14,15,16], and possibly exposure to formaldehyde [17]. Possible clusters of ALS have also been described amongst soccer players sharing the same environmental risks and occupational activities including frequent head trauma [18,19,20,21,22,23,24], but recent reports have also refuted specific links of head trauma to ALS [25,26,27]. Other forms of physical activity have been debated and despite many incidental reports, specific links with ALS have been refuted [28]. Of the environmental triggers for ALS, the cyanobacteria-derived neurotoxin BMAA continues to attract attention because of its ability to cause neurodegeneration *in vitro* and its ubiquitous nature [29,30,31,32,33], providing support for a potential role in the etiology of sporadic ALS [29,34,35]. 

## 2. History of Guam and Initial Theory of BMAA

When the United States recaptured the Marianas Islands in 1944 from Japan, they found an extremely high incidence rate of ALS and ALS-like conditions (ALS/ Parkinsonism dementia complex, ALS/PDC), particularly in Guam, where in 1954 it was estimated to be 50–100× higher than the worldwide rate [36,37,38]. Recent investigations suggest that the incidence and prevalence rates have declined considerably over the last 4 decades to levels of 7 per 100,000 that approach rates described in the rest of the world [39]. The disease also appears to have evolved over time to predominantly present clinically as parkinsonism and dementia rather than ALS. The chief factor responsible for the declining incidence appears to be ethnographic changes, social and ecological, brought about by the rapid westernization of Guam [39]. The change suggests that the cause of the disease is not genetic but rather environmental [39]. Early researchers suspected that something in cycad seeds, a dietary staple used by the Chamorro indigenous people to make flour, might be responsible for ALS/PDC [40]. The discovery of a neurotoxic non-protein amino acid, beta-N-methylamino-L-alanine (BMAA) in cycad flour suggested that this might be the particular cause of ALS/PDC [34,41]. BMAA in cycad seeds is derived from symbiotic cyanobacteria in coralloid roots of *Cycas* *micronesica* or possibly also from the cycad plant itself [42,43]. BMAA is mainly concentrated in proteins and was consumed by Chamorro through multiple dietary sources, including cycad flour, flying foxes (a type of fruit bat), and other animals that fed on cycad seeds [42,44,45,46]. 

## 3. BMAA in Brain Tissue

In the last ten years, Cox and colleagues have demonstrated that BMAA in cycad seeds is derived from symbiotic cyanobacteria in coralloid roots of *Cycas* *micronesica* and that BMAA in cycad flour is mainly concentrated in proteins. They were able to demonstrate that consumption of cycad flour, flying foxes, and other animals that fed on cycad seeds by the indigenous Chamorro people led to bio-concentration of protein-bound BMAA up the food chain, leading to the accumulation of BMAA in the brains of Chamorro patients with ALS/PDC (mean concentration of BMAA 627 µg/g) [34,42,44,45,46,47]. BMAA has also been detected in the brains of Canadian patients with Alzheimer’s disease (mean concentration of BMAA 107 µg/g) but not in control patients without neurological disease [46]. This latter finding has recently been confirmed by a second group (mean concentration of BMAA in Alzheimer’s brains 214 µg/g) and extended to show that BMAA is accumulated in the brains of US ALS patients (mean concentration of BMAA 268 µg/g), but not in those of patients with Huntington’s disease, a genetic neurodegenerative disease (mean concentration of BMAA 11 µg/g) or non-neurological controls (mean concentration of BMAA 41 µg/g) [35]. Huntington’s disease is purely a genetic neurodegenerative disease, so the inability to detect BMAA in these specimens is important as it suggests that BMAA does not occur as a byproduct of neurodegeneration.

## 4. Molecular Mechanisms of BMAA

Motor neurons are indisputably the largest and longest cells in the body making them vulnerable to any metabolic or external perturbation. Even fast retrograde and anterograde transport is a slow process in these very long axons moving at 100 mm/day. Because of the length of these cells, under the best of circumstances, it takes as much as two weeks for the fastest moving particles to go from the endplate to the cell body. Any small perturbation to transport or cellular hemostasis by an environmental toxin could have catastrophic effects. BMAA binds directly to NMDA and AMPA/kainate receptors and binding is enhanced when the BMAA is carbamated, producing a molecule that closely resembles glutamate [48,49]. BMAA has been found to induce selective motor neuron (MN) loss in dissociated mixed spinal cord cultures at concentrations of approximately 30 µM [49], significantly lower than those previously found to induce widespread neuronal degeneration and supporting the hypothesis that BMAA may contribute to the selective MN loss in ALS/PDC [49,50]. Lobner *et al.* have shown that the mechanism of neurotoxicity is actually three-fold; it involves not only direct action on the NMDA receptor, but also activation of glutamate receptor 5 (mGluR5) and induction of oxidative stress [51]. More recently Liu *et al.* [52] found that BMAA inhibits the cystine/glutamate antiporter system Xc^-^ mediated cystine uptake, which in turn leads to glutathione depletion and increased oxidative stress [52]. In cyclical-like pattern, BMAA appears to drive glutamate release via system Xc^-^ which induces toxicity through activation of the mGluR5 receptor. These multiple mechanisms of BMAA toxicity may account for its ability to induce complex neurodegenerative diseases. As pointed out by Bradley and Mash [34], although a single toxin triggering three different neurodegenerative diseases seems unusual, we know that mutations in the progranulin gene can result in ALS, AD, PD and PSP phenotypes in North American patients [53,54,55].

Conceivably, BMAA could be incorporated into proteins and subsequently lead to protein misfolding. The mechanism of BMAA incorporation into protein is not yet known, but a large fraction of BMAA is protein bound (60 to 130 fold greater quantity) compared to what has been recovered from the free amino acid pool [46]. It is also known that amino acid analogues can be incorporated into proteins and can alter cell function [56]. It is not known if BMAA causes misfolding, but there is literature showing the misincorporation of even the regular 20 amino acids, as with disruption of translational fidelity through the use of low levels of mischarged transfer RNAs (tRNAs) [57]. This misincorporation is associated with protein misfolding in terminally differentiated neurons [57]. The incorporation of BMAA into protein serves as a potential reservoir for future release of the toxin into cells [46].

Although there are many examples where BMAA displays neurotoxicity, no good animal model exists as of yet [58]. BMAA has been shown to cause motor neuron damage *in vivo* in brine shrimp (*Artemia* *salina* [59]), protozoa (*Nassula* *sorex* [59]), fruit flies (*Drosophila melanogaster* [60]), and fish (*Danio* *rerio* [59,61]). For a meta-analysis of BMAA neurotoxicity in other vertebrates, including primates, see Karamyan and Speth [58].

The hypothesis that BMAA may be a cause of ALS has its detractors. Snyder *et al.* reported being unable to detect BMAA in the brains of patients with Alzheimer’s disease and Guam ALS/PDC [62] and it has been pointed out that BMAA did not cause neurodegeneration in mice within their model system [62,63]. These observations have been criticized as being based on their own new assay methods rather than on the established AQC-derivatization assay and for not hydrolyzing the assayed material, thereby failing to release and measure the major portion of BMAA that is protein-bound [32,34,58]. Later papers from this same research team were able to find BMAA in the brain tissues of mice fed BMAA after they developed more sensitive analytical methods [64]. For whatever reason, the mouse model may be a poor model to demonstrate the neurotoxicity of BMAA and species-specific and allometric considerations are essential in these types of studies [58]. Despite these negative findings, the review by Karamyan and Speth [58] concluded that the neurotoxicity of BMAA was supported by almost all *in vivo* studies and is strongly associated with motor function.

## 5. Potential Exposures to and Bioaccumulation of BMAA

The production of BMAA is not confined to cycad seeds, and cyanobacteria species capable of producing BMAA are ubiquitously found throughout the world. In some locations, cyanobacteria are directly consumed by people and samples of these cyanobacteria have been shown to harbor BMAA. People from the Peruvian highlands collect cyanobacterial colonies of *Nostoc* *commune* (with a mean BMAA concentration of 10 µg/g) and eat them directly, sell them in markets, and add them to salads, soups, meat dishes and picante [65]. Likewise, in China, *fa* *cai* soup made with *Nostoc* *flageliforme* (with variable concentrations of BMAA ranging from not detectable to 0.66 µg/g) is eaten as a soup during New Year’s celebrations [66]. Unfortunately, epidemiological and human tissue analyses in these areas are lacking and higher incidence rates of ALS and ALS-like diseases are unknown. 

Cyanobacterial blooms can occur in both fresh water and marine water bodies, giving the potential for human exposure through direct consumption of water, recreational activities, and contaminated foodstuffs. In the United Kingdom, BMAA was prevalent in twelve water bodies used for either drinking water, recreation, or both [67]. BMAA was found within freeze-dried cyanobacteria collections obtained from these British water bodies as both a free amino acid (ranging from not detected to 276 µg/g) and as a protein associated amino acid (ranging from 6 to 48 µg/g). Likewise, BMAA has been indentified in scum material collected from urban waters in the Netherlands where cyanobacterial blooms are prevalent [68]. Recreational activities, such as swimming and water-skiing, are a potential source of exposure to BMAA in areas with cyanobacteria blooms; aerosol-borne exposure to another cyanobacteria toxin, microcystin, has already been documented [69,70]. In a recent article, Esterhuizen *et al.* [71] showed that free BMAA can be released during the collapse of cyanobacterial blooms. This process combined with cellular turnover creates a latent source of the non-protein amino acid for bioaccumulation and biomagnification in aquatic ecosystems. Rapid uptake of significant amounts of BMAA was observed in the macrophyte *Ceratophyllum* *demersum* [71]. Following accumulation of free BMAA, protein association was observed which suggests potential bioaccumulation by aquatic macrophytes and offers a possibility of phytoremediation for BMAA removal [71]. This also suggests a potential route for human exposure to BMAA if it is shown to apply more broadly to plants intended for human consumption. There is evidence to show that human exposure to other toxins of cyanobacterial origin occurs in this manner [72]. Bioaccumulation of BMAA is undoubtedly occurring within other organisms outside of the Guam ecosystem. 

Recently, Brand *et al.* [73,74] found that BMAA is bio-concentrated in crustaceans, mollusks, and certain fish, particularly those that feed on the benthos. This study provides one plausible environmental source of the BMAA identified in the South Florida ALS and Alzheimer’s disease patients described by Pablo *et al.* [35]; however, the very important correlative demographics and behaviors of individual patients are lacking at this time. The study by Brand *et al.* [74] has recently been confirmed in a second marine ecosystem in the Baltic Sea where the same pattern of high concentrations of BMAA was observed in bottom dwelling fish species and in filter-feeding mollusks [31]. Since massive blooms of cyanobacteria (e.g., *Nodularia*) are a common occurrence in the Baltic Sea because of its brackish waters and many organisms from the Baltic Sea are consumed by the surrounding human populations, this represents a concern for human health. Bioaccumulation of BMAA in marine environments is thus entirely feasible [75], but not demonstrated unequivocally as of yet. Caller *et al.* originally identified 9 ALS patients (now 11 ALS patients) who lived near Lake Mascoma in Enfield, NH, an incidence of sporadic ALS that is as much as 25 times the expected incidence for New Hampshire [76]. They suggested that the high incidence of ALS in this potential cluster might be related to a cyanobacteria toxin exposure from frequent cyanobacterial blooms in the lake. Blooms of cyanobacteria are found in many New Hampshire lakes each year and are capable of producing any number of toxins including BMAA [76]. Postulated mechanisms of BMAA acquisition include consuming contaminated fish, ingestion of lake water or possibly infiltration of lake water containing BMAA into artesian wells. Another postulated route of cyanotoxin exposure is inhalation of aerosolized toxins through wave action, as can occur with brevetoxins [77,78,79,80]. Besides wind and environmental factors, recreational activities in water bodies containing cyanobacterial blooms could potentially generate aerosolized cyanotoxins [70]. On an interesting note, cyanobacteria are major components of desert cryptobiotic soil crusts and BMAA was found in the desert crust of the Arabian Peninsula, where high winds constantly aerosolize the sand. Military activities in the Arabian Peninsula also created significant dust and more cases of ALS have been recorded in military personnel serving in this area during Gulf War I (Persian Gulf War) than in military personnel who were trained but not deployed [81]. 

## 6. Multiple Exposures to Cyanobacterial Toxins

Cyanobacteria are ubiquitous and produce a number of neurotoxins besides BMAA, including saxitoxin and anatoxin-a. Some species also produce the liver toxin microcystin which has been linked through careful epidemiological studies to liver disease and hepatocellular carcinoma [82,83,84,85]. BMAA has been shown to be co-produced with microcystin and other potent cyanobacterial toxins [67,86]. In addition, BMAA co-occurs with the neurotoxic isomer 2,4-diaminobutyric acid (2,4-DAB) in cycads and cyanobacteria [68,81,87,88]. It is established that not all cyanobacterial taxa are capable of producing some of the better characterized hepatic and neurologic toxins like microcystin, anatoxin-a, anatoxin-a(s), saxitoxin, and cylindrospermopsin. But, it is unclear at present whether all cyanobacteria are capable of producing BMAA under favorable conditions, and whether co-exposure to BMAA and other cyanobacterial toxins might potentiate the development of neurodegenerative disease. It is entirely possible that a synergistic effect could be created by exposure to more than one neurotoxin at a time. Lobner *et al.* demonstrated that *in vitro*, BMAA at concentrations as low as 10μM potentiate neuronal damage caused by other insults [51].

## 7. Nutritional Status of Persons Exposed to BMAA

Lathyrism or neurolathyrism is a neurological disease of humans and domestic animals, caused by consuming the legume *Lathyrus* *sativus*. The consumption of large quantities of *Lathyrus* * sativus* (400g/day) containing high concentrations of the glutamate analogue neurotoxin β-*N*-oxalyl-amino-L-alanine (BOAA) causes a spastic paraparesis [89]. Lathyrism occurs during periods of malnutrition and is often triggered by physical exhaustion. Adverse environmental conditions brought on by drought, flood, pestilence and war have forced populations to rely almost exclusively on food resistant to these adverse conditions such as the *Lathyrus* *sativus* legume; a situation that leads to the development of Lathyrism [90]. Supplemental food-aid appears to reduce the incidence of neurolathyrism in areas of drought and malnutrition [91]. Jahan and Ahmad discovered that experimental neurolathyrism could be produced in guinea pigs and primates that needed an external supply of ascorbic acid by making them subclinically deficient in ascorbic acid and feeding them the seeds of *Lathyrus* *sativus* or extracts thereof [92]. Autoclaving the seeds of the *Lathyrus* *sativus* with lime removed the toxin [92]. As with BOAA, it is postulated that BMAA could be more harmful to malnourished individuals [93]. ALS/PDC was first discovered in Guam and peaked following World War II at a time when the indigenous Chamorro people were malnutritioned. By the time it was documented, dietary intake was probably back to near normal but years of malnutrition may have contributed to the overall peak of disease that occurred later. 

## 8. Latency Effects of BMAA

The lag time between BMAA exposure and onset of neurodegeneration is not known, however, considerable insight can be found from the analysis of migrants to and from Guam. Twenty-eight Chamorro migrants from Guam developed ALS/PDC after periods of absence ranging from 1 to 34 years [94]. These data suggest that if environmental factors are responsible the latency period for disease development may be over three decades. Estimates of crude mortality rates from ALS for these migrants are at least three times as high as rates noted for the United States population, yet more than four times lower than the ALS crude mortality rates for non-migrant Chamorros living in Guam during the twenty years prior to the epidemiological study [94] suggesting that the exposure the migrants were subjected to occurred in Guam. When considering cases of progressive neurologicial disease among Filipino migrants to Guam, ten Filipino migrants to Guam were reported with ALS 1 to 32 years after their arrival; two migrants developed parkinsonism-dementia 13 and 26 years after arrival, and seven additional patients developed what was considered more classic PD 5 to 24 years after their migration to Guam [95]. Ten children born on Guam of Filipino and Chamorro parents also developed ALS and six developed PD [95]. The average annual crude mortality rate for ALS among Filipino migrants was estimated to be six times higher than those living in the continental United States but half the rate observed among Chamorros living on Guam during the same period [95]. Combined, these data suggest that environmental exposure precedes neurological symptoms by possibly decades and that some people are more susceptible to disease than others due to interactions between genes and environmental exposure. 

## 9. Conclusions

Cyanobacteria are ubiquitous and many of the species examined so far have been shown to produce the neurotoxic non-protein amino acid BMAA. Since human exposure to BMAA appears to be widespread, it has the potential to be a major environmental factor capable of causing ALS and other neurodegenerative diseases throughout the world [34]. The literature is full of convincing spatial clusters of ALS and reports of conjugal ALS [24,76,96,97,98,99,100,101,102,103]. A careful evaluation of these clusters in relation to potential environmental sources of ALS would be very important as would evaluation of brain and spinal cord tissues for the presence of BMAA. Although the link between BMAA and sporadic ALS awaits a primate model of BMAA-induced progressive neurodegeneration for better confirmation [58], multi-disciplinary studies by investigators at a variety of institutions indicate that BMAA should be more carefully investigated as a trigger for ALS acting through gene-environment interactions. 
